# Biomechanical Contributions to Macrophage Activation in the Tumor Microenvironment

**DOI:** 10.3389/fonc.2020.00787

**Published:** 2020-05-20

**Authors:** Erica J. Hoffmann, Suzanne M. Ponik

**Affiliations:** ^1^Department of Cell and Regenerative Biology, Wisconsin Institutes for Medical Research, University of Wisconsin-Madison, Madison, WI, United States; ^2^University of Wisconsin Carbone Cancer Center, University of Wisconsin-Madison, Madison, WI, United States

**Keywords:** macrophage activation, breast cancer, extracellular matrix, tumor microenvironment, integrins, collagen, mechanosensing

## Abstract

Alterations in extracellular matrix composition and organization are known to promote tumor growth and metastatic progression in breast cancer through interactions with tumor cells as well as stromal cell populations. Macrophages display a spectrum of behaviors from tumor-suppressive to tumor-promoting, and their function is spatially and temporally dependent upon integrated signals from the tumor microenvironment including, but not limited to, cytokines, metabolites, and hypoxia. Through years of investigation, the specific biochemical cues that recruit and activate tumor-promoting macrophage functions within the tumor microenvironment are becoming clear. In contrast, the impact of biomechanical stimuli on macrophage activation has been largely underappreciated, however there is a growing body of evidence that physical cues from the extracellular matrix can influence macrophage migration and behavior. While the complex, heterogeneous nature of the extracellular matrix and the transient nature of macrophage activation make studying macrophages in their native tumor microenvironment challenging, this review highlights the importance of investigating how the extracellular matrix directly and indirectly impacts tumor-associated macrophage activation. Additionally, recent advances in investigating macrophages in the tumor microenvironment and future directions regarding mechano-immunomodulation in cancer will also be discussed.

## Introduction

Macrophages are an innate immune cell type found in all tissues of the body with multiple functions. Tissue resident pools of macrophages arise from embryonic tissues during development, and are critical for normal tissue morphogenesis (1). During homeostasis, tissue macrophages are maintained primarily through local proliferation. In chronic inflammatory processes such as cancer, hematopoietic derived monocytes circulate through the blood and infiltrate tissues where they terminally differentiate into macrophages to, in part, replenish resident pools as well as increase macrophage levels for the remediation of infection or structural damage (2). Macrophages display a spectrum of opposing yet complementary behaviors depending on the signals they receive from the local microenvironment (3). Traditionally, macrophage activation has been characterized using a dichotomous spectrum, with the two extremes being “classically activated” or pro-inflammatory macrophages and “alternatively activated” or pro-remodeling, immunosuppressive macrophages. Classically activated macrophages (termed M1) phagocytize microbes and secrete cytokines such as interleukin 6 (IL-6), TNF-α, and IL-1β, as well as nitric oxide (NO) and reactive oxygen species during host defense in response to stimulation by interferon-γ (IFN-γ) and toll-like receptor ligands, including bacterial lipopolysaccharide (LPS). Alternatively activated macrophages (termed M2) are stimulated primarily by the T_h_2 cytokines IL-4 and IL-13 and facilitate extracellular matrix (ECM) remodeling, blood vessel formation, and dampen immune activation by secreting cytokines such as IL-10 and TGF-β (4, 5). In recent years it has become apparent that the dichotomous M1/M2 model is an oversimplification of the behavioral spectrum of macrophages, with many unique transcriptional profiles being identified in response to differing activation signals (6). As such, it is now recommended to denote macrophage states by the activating stimulus (e.g., M_LPS+IFNγ_ or M_IL4+IL13_) (7). Macrophage activation states have been characterized extensively in murine and *in vitro* models. However, the exact genetic profiles and functional outputs, such as NO production (8, 9), for example, differ from human macrophage states and the relevance of murine studies to human macrophage biology is still under debate. Nonetheless, both major macrophage phenotypes are required for maintaining tissue homeostasis, but each, respectively, can play a role in the pathogenesis of diseases including atherosclerosis and cancer (10).

## Macrophages and the Extracellular Matrix in Cancer

In cancer, macrophages infiltrate the tumor microenvironment (TME) in response to tumor-secreted chemotactic signals and exhibit a tumor-supportive phenotype similar to the M2 phenotype. High macrophage infiltration has been associated with a poor prognosis and increased rates of metastasis in several cancer types, as tumor-associated macrophages (TAMs) can facilitate blood vessel formation to support expanding tumor growth and aid tumor cell intravasation into vasculature (5, 11–13). Much work has been done to characterize soluble factors present in the TME that recruit and influence macrophage behavior (14), however less is known about how the mechanical properties of tumor ECM impact macrophage recruitment, activation, and cytokine secretion.

Many cancers, including breast cancer, exhibit aberrant deposition, and organization of extracellular matrix proteins surrounding a tumor (15–18). The ECM is comprised of several fibrous and non-fibrous proteins including collagens, laminins, fibronectin, and others that are deposited and organized into a stromal meshwork that supports cellular growth and migration. Indeed, dense breast tissue is a strong and prevalent risk factor for the development of invasive breast cancer and is associated with excess collagen deposition and tissue stiffness (19–23). Recent studies demonstrate that even in healthy patients, mammographically dense tissue increases pro-tumor inflammation and overall immune infiltration, including CD68+ macrophages and CD20+ B lymphocytes surrounding the vasculature, which may be part of the underly mechanism driving this risk of developing breast cancer (24). In breast cancer patients, the reorganization of collagen that accompanies tumor progression results in aligned fiber bundles at the tumor-stromal boundary and, importantly, this signature of collagen predicts disease outcome (18, 25). Along these lines, in invasive ductal carcinoma biopsy tissue, the association of anti-inflammatory CD163+ macrophages and aligned collagen fibers is predictive of poor patient outcome (26). Macrophages have been shown to play a role in matrix organization through the secretion of matrix metalloproteinases to degrade and reorganize the matrix as well as aid in tumor cell migration (27). Moreover, tumor associated macrophages have been shown to facilitate the deposition of aligned collagen fibers during tumor development (28, 29).

**Figure 1:**
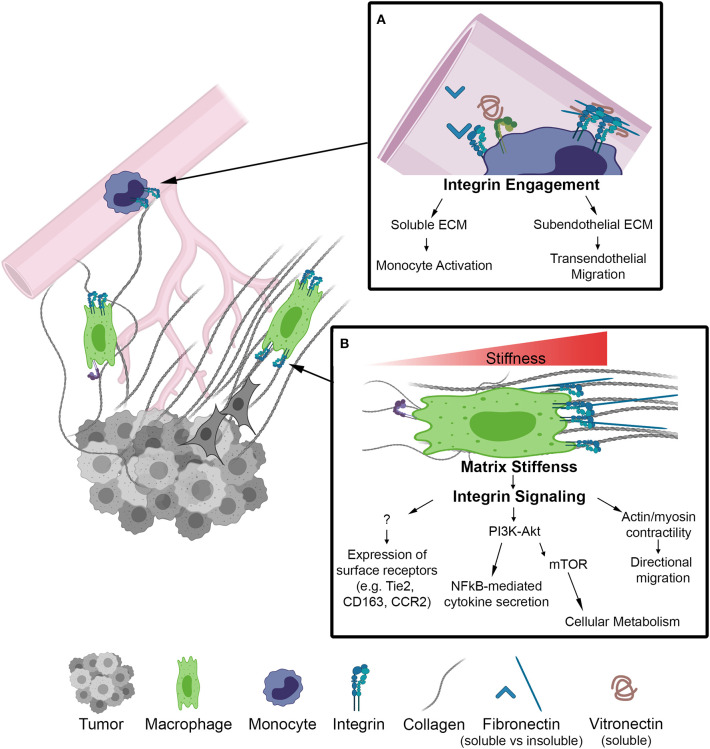
Schematic of biophysical cues from the ECM to activate integrin signaling on macrophages in the TME. **(A)** Inset depicts integrins on the surface of monocytes within the lumen of a blood vessel. Integrin engagement activates monocytes in circulation and facilities transendothelial migration into the TME. **(B)** Macrophage localized in a region of increasing matrix stiffness. Matrix stiffness results in integrin clustering and focal adhesion signaling. Downstream of integrins there is an increase in the PI3K/Akt pathway to activate NF-kB transcriptional activity as well as actin/myosin generated cellular contractility leading to directional migration. Further investigation is required to determine whether integrin signaling regulates other markers of macrophage activation.

As monocytes circulate toward tumor signals they encounter soluble plasma matrix proteins, such as fibronectin and fibrinogen, known to be upregulated in breast cancer patients and associated with poor prognosis (30, 31). The binding of these ECM proteins to adhesion receptors on the surface of macrophages promote inflammatory and tumor-promoting macrophage activation (32–34) (Figure 1A). Within tumor stroma, collagen along with fibronectin and laminin have been shown to promote tumor cell proliferation, angiogenesis, and dissemination (35, 36). Alterations in ECM organization and composition in the tumor microenvironment result in increased matrix stiffness, primarily localized to the invasive front of breast tumors. These stiff regions are enriched in aligned collagen fibers, tumor-activated macrophages (CD163+) and the activated form of β1-integrin (23). Similarly, accelerated tumor progression was accompanied by an overall increase in macrophages and tumor cytokines, including CCL2, in a collagen-dense mammary tumor model compared to controls (37, 38). Moreover, CCL2 recruits Tie2 expressing macrophages to facilitate early tumor cell dissemination (39). This process involves a mechanism by which macrophages lead tumor cells through reciprocal chemokine signaling along collagen coated substrates and toward vascular endothelium *in vitro*. Importantly, the same mechanism of macrophage-tumor cell migration has been observed *in vivo*, where macrophage-tumor cell trafficking can be visualized along collagen fibers (40, 41). Together, these studies suggest that matrix stiffness increases CCL2 levels, which in turn recruits specific macrophage populations that interact with collagen fibers and facilitate tumor cell dissemination. Thus, it is becoming clear that macrophages are sensitive to changes in the ECM and their mechanical environment, however the causal link between ECM biophysical properties and the functional activation of TAMs *in vivo*, in animal models as well as in humans, is still unclear.

## Mechanical Regulation of Macrophage Polarization

Growing appreciation for biophysical cues from the extracellular matrix to drive cellular phenotypes has led to a large body of work demonstrating that ECM topography, composition, stiffness, and other mechanical loading modalities are capable of modulating macrophage function *in vitro*. The field of macrophage mechanobiology has largely stemmed from the biomaterials and implant fields. These fields have found that changing surface topography by increasing surface roughness generally results in increased macrophage adhesion and alterations in cytokine secretion, but the mechanisms by which roughness impacts macrophage responses depends on the method and the macrophage cell types used for investigation (42–44). Other studies have demonstrated that substrate stiffness, which is associated with enhanced breast tumor progression, is another mechanical aspect of the ECM that can influence macrophage behavior (Figure 1). Increasing the stiffness of biologic and engineered substrates resulted in increased migration of unstimulated macrophages, and inflammatory macrophage cytokine production (45, 46). However, it should be noted that these increases are accompanied by altered integrin expression levels as well as increased myeloid differentiation response protein 88 (MyD88)-dependent NF-κB activation through TLR activation, and that NF-κB has been shown to regulate anti-inflammatory gene expression as well (47). Additionally, these effects are independent of collagen I and laminin stimulation and may be the result of cytoskeletal signaling rather than integrin engagement.

Changes in cell shape and the cytoskeleton are also frequently observed with increasing substrate stiffness and can in themselves alter macrophage activation. In general, M1 macrophages are uniformly spread, with a circular morphology (45, 48). Inflammatory activation is inhibited when bone marrow derived macrophages (BMDMs) are confined to small pores rather than allowed to spread freely, through a mechanism involving actin dynamics and signaling through the MRTF-A-SRF complex (49). However, elongation of macrophages produces a different phenotype. Macrophages elongated on 2-D engineered nano-substrates consistently correlates with anti-inflammatory gene expression profiles across a variety of surfaces and cell lines at late time points (>24 h) and when cells are allowed to spread along wider grooves (>450 nm widths). Furthermore, macrophage elongation increases expression of adhesion receptors, actin-based contraction and enhances activation by IL-4/IL-13, while preventing elongation attenuates these cytokines' ability to activate the macrophages (45, 48, 50, 51).

In many of these studies, it appears that mechanical stimuli may work in conjunction with soluble factors to induce macrophage activation. Nevertheless, mechanical stimulation likely plays an equally important role in priming macrophages to become activated toward a specific phenotype, however the exact cellular mechanisms and intracellular signaling pathways that mediate this still require further investigation. Therapeutically, there is potential to modulate macrophage behavior via mechanical regulation, however the application of this knowledge in the context of cancer remains limited, as more work is required to characterize the mechanical dynamics present within the TME. Presently, there are few therapies that directly target ECM stiffness or organization. Therefore, understanding how the ECM can modulate the activity of soluble signals on macrophages in the TME, through adhesion receptors and the cytoskeleton for example, may provide insights into improving existing therapies that target cytokine and growth factor signaling.

## Integrin Adhesion Signaling in Macrophage Activation

### Overview

As previously eluded to, mechanical cues from the ECM can be detected by macrophages through the integrin family of heterodimeric adhesion receptors, and many integrins are differentially expressed by classically and alternatively activated macrophages (45). Integrins consist of an alpha and beta subunit. Each alpha and beta combination has a unique binding affinity for certain matrix proteins, however each integrin often has multiple ECM ligands. Upon ligand binding, integrins transduce signals inside of the cell via adapter proteins such as focal adhesion kinase (FAK), talin, vinculin, and others that couple integrins to the cytoskeleton (outside-in signaling) (52). Changes in cytoskeletal organization have a direct impact on several transcription factors, including MRTF-A, YAP and NF-κB, which facilitate changes in gene transcription that are potentially related to macrophage function. Several integrins are expressed by macrophages (Table 1), the most common being the β2 family of integrins which are unique to leukocytes. Although integrin signaling has traditionally been overlooked when investigating macrophage activation, several studies have demonstrated that integrin-ECM adhesion initiates signaling pathways that can in fact influence macrophage activation. Based on these studies the concept emerges that biophysical cues from the ECM regulate macrophage activation, in part, through integrin engagement and signaling (Figure 1).

**Table 1:** Integrins expressed on the surface of murine macrophages.

	**Integrin**	**ECM Ligands**	**Other ligands**	**Main functions**	**References**
*β2 Family*	**αLβ2** CD11a/CD18 LFA-1	None.	ICAM-1 ICAM-3 ICAM-2 ICAM-5 JAM-1	Endothelial transmigrationIntercellular adhesion	(53–56)
	**αMβ2** CD11b/CD18 Mac-1 CR3	Fibronectin Vitronectin Fibrinogen Laminins Collagens Cyr61	ICAM-1 ICAM-2 ICAM-3 iC3b Thrombospondin CD23 NIF LPS [for complete list please see (57)]	Migration Complement Receptor Type 3 Phagocytosis Trans-endothelial extravasation	(57–60)
	**αXβ2** CD11c/CD18 P150,95 CR4	Fibrinogen	ICAM-1 ICAM-4 CD23 LPS Thy-1 iC3b Plasminogen	Complement Receptor Type 4 Intercellular adhesion Fibrinogen adhesion	(60–67)
	**αDβ2** CD11d/CD18	Fibronectin Vitronectin Fibrinogen Cyr61	ICAM-3 Plasminogen P2-C	Migration Cell adhesion	(68–70)
*β1 Family*	**α2β1** *VLA-2* *CD49b/CD29*	Collagens Laminins	Echovirus 1	Migration Cell adhesion	(71–73)
	**α4β1** *VLA-4*	Fibronectin EMILIN1	VCAM-1	Migration Intercellular adhesion	(71, 74, 75)
	**α5β1** *VLA-5*	Fibronectin	RGD Sequences	Fibronectin receptor Migration	(71, 76, 77)
	**α6Aβ1** *VLA-6*	Laminin (not in macrophages, however) Fibronectin	–	Adhesion	(71, 78)
*β3 Family*	**αVβ3** *CD51/CD63*	Vitronectin Fibrinogen	VWF Thrombospondin RGD Sequences	Vitronectin receptor Adhesion	(58, 77, 79)
*β5 Family*	**αVβ5**	Vitronectin (Fibrinogen and Fibronectin, minimally)	MFG-E8	Phagocytosis Debris clearance	(80–82)

*Integrin names are listed using α and β chain nomenclature with commonly used alternative names listed underneath*.

### Effects of Integrin Activation on Macrophages

The αMβ2 integrin (also commonly referred to as CD11b/CD18 and Mac-1, among others) is the most promiscuous integrin of the β2 integrin family. It is also the most studied of the integrins expressed by macrophages, however its impact on macrophage activation remains disputed. In *Itgam*^−/−^ (αM deficient) mice, tumor growth and immunosuppressive cytokine mRNA levels are enhanced relative to wild type mice, whereas constitutive activation of the αM integrin by a point mutation knock in (C57BL/6 ITGA-M ^I332G^) inhibits tumor growth, despite increased IL-6 mRNA levels (83). In contrast, Han et al. argue that inflammatory cytokines are upregulated in *Itgam*^−/−^ mice (relative to *Itgam*^+/−^ control mice) when challenged with TLR ligands (84). However, this increase is measured from serum and global knockout of αM likely impacts other immune cell types, such as dendritic and natural killer cells, which could contribute to this finding. αMβ2 expression is upregulated in stiff, photo-induced cross-linked fibrin gels (45) and by the inflammatory stimuli LPS/ IFN-γ. Its expression is also inhibited by TGF-β, a protein that is abundant in the TME and may contribute to tumor-directed immune suppression (83). On the other hand, work by the Xuetao Cao group has shown that TLR-mediated αMβ2 activation, that leads to downstream Src and Syk activation, is capable of promoting alternative activation in murine macrophages via a IL-4-STAT6-Jak1 and MyD88-TRIF-Cbl-b mediated mechanism, respectively (84, 85). Additionally, lysyl oxidase (LOX)-mediated collagen crosslinking within the primary and pre-metastatic TME aids in the retention of myeloid cells expressing the αMβ2 integrin. The αMβ2+ macrophages secrete MMPs to continue to reorganize the ECM, further contributing to increased macrophage levels in primary and metastatic breast tumors (23, 86, 87).

In addition to αMβ2, collagen specific adhesion receptors have also been shown to mediate macrophage activation. The importance of macrophage adhesion to collagen is underscored by the fact that the ECM in human primary breast cancers contains higher levels of collagen (I, III, IV, XIII) compared to normal breast tissue (88, 89). The α2β1 integrin mediates macrophage migration and adhesion to type 1 collagen. A study by Cha et al. showed that α2β1, vinculin, PTK2, and the alternatively activated macrophage-associated marker CD206 are significantly upregulated by macrophages differentiated from THP-1 monocytes on hydrogels that allow for cell adhesion. Furthermore, this adhesion-mediated signaling augments the effects of IL-4 treatment. When α2β1 ligand binding is blocked with a neutralizing antibody, CD206 expression is significantly downregulated and cannot not be induced by the addition of IL-4, demonstrating that α2β1 engagement is important for alternative activation (90). Independent of soluble factors, it has also been shown that macrophages are able to sense mechanical deformations of the ECM from fibroblast contractions, and that these deformations alone are able to induce α2β1 mediated macrophage migration toward the fibroblasts (91). High numbers of cancer-associated fibroblasts are often observed in tumors, suggesting that cellular contractions from cancer-associated fibroblasts may dramatically deform the ECM to potentially aid the recruitment of α2β1-expressing TAMs locally. Moreover, scavenger receptor A (SR-A) and CD36 mediate macrophage adhesion to modified or denatured forms of type I and IV collagen, which are often found in inflammatory conditions (92–94). CD36 is upregulated in alternatively activated macrophages (95), and SR-A is upregulated by macrophages when co-cultured with cancer cells (96). Interestingly, SR-A expressing TAMs colocalize in the stroma of tumors with FAP+ cancer associated fibroblasts that cleave collagen fibers to enhance TAM retention via SR-A mediated adhesion (94). SR-A-mediated macrophage adhesion plays an important role in cancer, as demonstrated by the prevention of ovarian cancer progression in mice treated with SR-A inhibitors (96, 97).

Several other ECM protein ligands bind integrins expressed by macrophages. The β3 integrin is required for macrophage trans-endothelial migration on the ECM protein vitronectin. In human peripheral monocyte derived macrophages, ligand binding to αVβ3 integrins activates a PI3-K/Akt signaling cascade resulting in NF-κB mediated gene expression and pro-inflammatory cytokine secretion. Interestingly, this pathway is synergistically enhanced by LPS/TNF-α stimulation (98). In contrast, in murine BMDMs β3 expression was seen to be significantly higher in M_IL4+IL13_ macrophages compared to M_LPS+IFNγ_, and its knockdown resulted in increased TNF-α secretion relative to the non-treated control (45). Additionally, the β4 laminin binding integrin is upregulated on the surface of TAMs in triple negative breast cancer. In combination with TGF-β signaling, ligand binding to β4 leads to increased integrin clustering and adhesion to lymphovasculature, which aids tumor cell dissemination (99).

Integrins are critical for cellular migration, and while macrophages are capable of utilizing both amoeboid and mesenchymal modes of migration, certain integrins may enhance macrophage migration in parallel with chemotactic signals. Macrophages can sense increases in fibronectin within the TME via the α5β1 integrin (58). β1 binding to fibronectin can couple with CSF1R, a master regulator of macrophage function and survival, on the plasma membrane leading to CSF1R-mediated phosphorylation via SFK/FAK (100). CSF1R has been strongly implicated in the recruitment and regulation of tumor promoting activities of TAMs (101), and is necessary for macrophage migration on fibronectin (100). Some have suggested that inflammatory signaling is required to prime integrins into the active state, allowing for increased ligand binding and signal transduction responsible for gene transcription, and interactions between adhesion and cytokine receptors lends strength to this argument (58).

## Studying Macrophages *in vivo*

### Challenges

Many challenges still exist when investigating macrophage biology, both *in vitro* and *in vivo*. The inconsistent findings from many of the studies discussed here can potentially be attributed to differences in cell lines, surface chemistries, time points analyzed, and other variables, but nonetheless emphasize the important fact that commonly used macrophage cell lines and primary cells exhibit differing responses to identical stimuli, often making *in vitro* findings difficult to compare. This is true for both murine (4, 102, 103) and human (104, 105) cell sources. Additionally, there are many differences between human and murine macrophage biology, from surface marker expression to metabolic states, that can result in stark differences in functional output (106–108). Species specificity of macrophage cell types and the presence or absence of serum factors from humans vs. other species used in *in vitro* studies may also limit the applicability to human biology and therapeutic strategies. Thus, further studies are required to delineate murine and human macrophage responses, not only in mechanical studies.

Additional challenges exist when identifying the activation state of a macrophage, especially *in vivo*. Traditionally, phenotypes are identified using immunohistochemistry and transcriptional profiling, however these techniques require multiple markers to confirm an activation state and are most useful in *in vitro* or *ex vivo* studies at end stage time points. There is a great need for techniques to identify phenotypes through protein expression *in vivo*. While the use of genetically encoded fluorescent proteins to readout macrophage activation is possible, the use of multiple markers to confirm macrophage identity and the unintended effects of introducing exogenous proteins limits feasibility. Another area of concern, particularly in studies investigating mechanical regulation of macrophages is the fact that macrophages respond differently to substrates in 2-D compared to 3-D. Currently, most studies are performed using 2-D methods to investigate migration and activation. There is a great need for more studies investigating macrophages in 3-D, especially in the context of cancer, as it is more representative of the environment macrophages naturally reside in. It is imperative to improve methods of investigating macrophages in their native environments so as to minimize variances that arise from culture and experimental conditions, and to best elucidate the impact of the ECM on macrophages.

### Current Approaches

In order to observe macrophages in the tumor microenvironment, the field has recently turned to optical approaches such as positron emission tomography (PET), for example [reviewed extensively in (109)]. Rostam et al. have proposed image-based machine learning to identify phenotypes based on cellular morphology which, as described earlier, may provide some indication of phenotype (110). The availability of 3-D culture platforms to investigate macrophage–tumor cell interactions provide a tool kit to identify macrophage phenotype in more *in vivo*-like microenvironments (111–113). Using these platforms, one can take advantage of pharmacologic and optogenetic approaches to manipulate adhesion receptor activation and downstream signaling pathways involved in macrophage responses to biophysical cues from the ECM (114–116).

**Figure 2:**
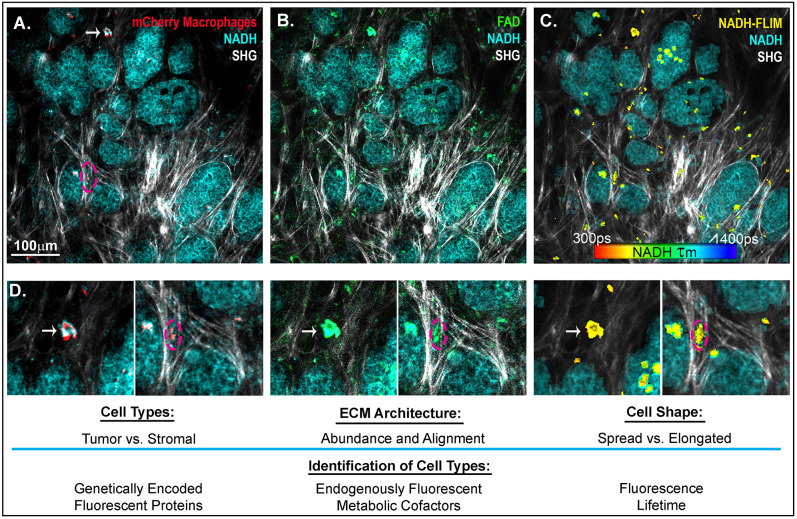
Intravital imaging of mammary carcinoma in a MMTV-PyMT mouse. 2-photon scanning laser microscopy allows for the *in vivo* observation of tumor cells (high in NADH intensity, 780 nm excitation), collagen fibers through second harmonic generation (890 nm excitation), and **(A)** Macrophages expressing the fluorescent mCherry protein under the CSF1-R promotor (C57BL/6-Tg(Csf1r-HBEGF/mCherry)1Mnz/J X B6.129P2-Lyz2^tm1(cre)Ifo^/J) (120) (1050nm excitation). **(B)** FAD^HI^ cells which depict primarily macrophage stromal cells (119). **(C)** NADH fluorescence lifetime overlay on mask of mCherry+ cells (color map of NADH τm lifetime). **(D)** Insets depict mcherry^+^ macrophages, which are FAD bright, spatially localized in the collagen rich stroma or within the tumor mass. Arrow indicates a macrophage spread in a collagen abundant region of the tumor stroma. Dashed outline depicts a macrophage elongated in an aligned region of collagen fibers at the boundary of a tumor nest.

In addition to PET and single-photon emission computed tomography (SPECT) (109), another technique, intravital imaging, utilizes small implanted imaging windows paired with confocal or multiphoton microscopy to visualize the spatial organization of tumor and stromal cell populations (117, 118). Cell-type-specific expression of proteins that are genetically fused with fluorescent tags, such as GFP or mCherry, as well as the endogenously fluorescent metabolic cofactors FAD^+^ and NADH (119) can be used to identify macrophage, tumor, and other cell types in the mouse (Figure 2). This technique has facilitated direct observation of macrophages interacting with and assisting tumor cells to intravasate into nearby vasculature, as well as tumor cell extravasation at distant metastatic sites (121, 122). While this approach provides detailed spatial and temporal resolution of cells in the TME, there is still a lack of validated signatures to fully identify and characterize macrophage phenotypes *in vivo*. One emerging signature of macrophage activation is the use of fluorescence lifetime. Fluorescence Lifetime Imaging Microscopy (FLIM) reports the time a fluorophore remains in the excited state before transitioning back to ground state, and differences in fluorescent lifetimes of NADH and FAD^+^ can indicate whether the cofactors are free or protein bound. Changes in the relative concentrations of bound vs. free NADH and FAD^+^ can provide information on metabolic states at the single cell level (123, 124). Within the TME, Szulczewski et al. demonstrated that stromal macrophages have a distinct NADH FLIM signature, allowing them to be distinguished from tumor cells (119). Along these lines, Alfonso-Garcia et al. show stark differences in the NADH fluorescence lifetime signatures in M_LPS+IFNγ_ and M_IL4+IL13_ induced BMDMs *in vitro* (125), thus warranting further investigation into the use of FLIM to identify macrophage activation *in vitro* and *in vivo*. In addition to endogenous and genetically expressed fluorescence, ported mammary imaging windows that feature a needle inserted through the window base have been used to inject fluorescently conjugated antibodies. This methodology provides an opportunity for real-time visualization of the localization and relative abundance of cell type specific proteins, such as macrophage activation markers and integrins.

## Conclusion

Taming tumor-associated macrophages has been a long-time goal for cancer therapy, and much work remains to fully understand the crosstalk between macrophages and the tumor microenvironment. While a causal mechanistic link between biomechanical properties of the ECM and macrophage activation has yet to be fully established *in vivo*, here we highlight studies that investigate the relationship and crosstalk between biophysical properties of the ECM and macrophage activation. Further investigation into downstream signaling pathways activated by integrin ligand binding and mechanical stimuli is necessary to identify potential therapeutic interventions to shift TAMs away from a tumor promoting phenotype. One expanding area is the use of metabolic reprogramming to shift macrophage phenotypes. Classically and alternatively activated macrophages favor differing metabolic mechanisms, and differences in the fluorescence lifetime signatures of metabolic cofactors lends support to the use of metabolism as a phenotypic marker. Moreover, integrin activation through the α2β1 integrin can induce activation the PI3K-Akt pathway (126), and macrophage metabolism is strongly regulated by PI3k-Akt-mTOR signaling which can prime macrophages toward either activation state depending on confounding biochemical stimuli in the TME such as hypoxia or IL-4 (127). Metabolism provides an attractive target for manipulation, as it is highly sensitive and fast responding to changes inside and outside the cell, critical characteristics for macrophages to alter their activation in an inducible and reversible manner.

## Author Contributions

EH wrote the first draft of the manuscript. All authors contributed to manuscript and figure conceptualization and design, revision, read and approved the submitted version.

## Conflict of Interest

The authors declare that the research was conducted in the absence of any commercial or financial relationships that could be construed as a potential conflict of interest.

## References

[1] Ingman WV Wyckoff J Gouon-Evans V Condeelis J Pollard JW. Macrophages promote collagen fibrillogenesis around terminal end buds of the developing mammary gland. Dev Dyn. (2006) 235:3222–9. 10.1002/dvdy.20972 17029292

[2] Movahedi K Van Ginderachter JA. The ontogeny and microenvironmental regulation of tumor-associated macrophages. Antioxid Redox Signal. (2016) 25:775–91. 10.1089/ars.2016.6704 27020982

[3] Smith TD Tse MJ Read EL Liu WF. Regulation of macrophage polarization and plasticity by complex activation signals. Integr Biol. (2016) 8:946–55. 10.1039/c6ib00105j 27492191 PMC5148158

[4] Gundra UM Girgis NM Ruckerl D Jenkins S Ward LN Kurtz ZD. Alternatively activated macrophages derived from monocytes and tissue macrophages are phenotypically and functionally distinct. Blood. (2014) 123:e110–22. 10.1182/blood-2013-08-520619 24695852 PMC4023427

[5] Chittezhath M Dhillon MK Lim JY Laoui D Shalova IN Teo YL. Molecular profiling reveals a tumor-promoting phenotype of monocytes and macrophages in human cancer progression. Immunity. (2014) 41:815–29. 10.1016/j.immuni.2014.09.014 25453823

[6] Xue J Schmidt SV Sander J Draffehn A Krebs W Quester I. Transcriptome-based network analysis reveals a spectrum model of human macrophage activation. Immunity. (2014) 40:274–88. 10.1016/j.immuni.2014.01.006 24530056 PMC3991396

[7] Murray PJ Allen JE Biswas SK Fisher EA Gilroy DW Goerdt S. Macrophage activation and polarization: nomenclature and experimental guidelines. Immunity. (2014) 41:14–20. 10.1016/j.immuni.2014.06.008 25035950 PMC4123412

[8] Schneemann M Schoeden G Macrophage biology and immunology: man is not a mouse. J Leukoc Biol. (2007) 81:579–9. 10.1189/jlb.1106702 29350850

[9] Gross TJ Kremens K Powers LS Brink B Knutson T Domann FE. Epigenetic silencing of the human NOS2 gene: rethinking the role of nitric oxide in human macrophage inflammatory responses. J Immunol. (2014) 192:2326–38. 10.4049/jimmunol.1301758 24477906 PMC3943971

[10] Wynn TA Chawla A Pollard JW. Macrophage biology in development, homeostasis and disease. Nature. (2013) 496:445–55. 10.1038/nature12034 23619691 PMC3725458

[11] Bingle L Brown NJ Lewis CE. The role of tumour-associated macrophages in tumour progression: implications for new anticancer therapies. J Pathol. (2002) 196:254–65. 10.1002/path.1027 11857487

[12] Zhang QW Liu L Gong CY Shi HS Zeng YH Wang XZ. Prognostic significance of tumor-associated macrophages in solid tumor: a meta-analysis of the literature. PLoS ONE. (2012) 7:e50946. 10.1371/journal.pone.0050946 23284651 PMC3532403

[13] Lin Y Xu J Lan H. Tumor-associated macrophages in tumor metastasis: biological roles and clinical therapeutic applications. J Hematol Oncol. (2019) 12:76. 10.1186/s13045-019-0760-3 31300030 PMC6626377

[14] Hao NB Lü MH Fan YH Cao YL Zhang ZR Yang SM. Macrophages in tumor microenvironments and the progression of tumors. Clin Dev Immunol. (2012) 2012:948098. 10.1155/2012/948098 22778768 PMC3385963

[15] Flier JS Underhill LH Dvorak HF Tumors: wounds that do not heal. N Engl J Med. (1986) 315:1650–9. 10.1056/NEJM198612253152606 3537791

[16] Walker C Mojares E Hernández AD. Role of extracellular matrix in development and cancer progression. Int J Mol Sci. (2018) 19:3028. 10.3390/ijms19103028 30287763 PMC6213383

[17] Lochter A Bissell MJ. Involvement of extracellular matrix constituents in breast cancer. Semin Cancer Biol. (1995) 6:165–73. 10.1006/scbi.1995.0017 7495985

[18] Conklin MW Eickhoff JC Riching KM Pehlke CA Eliceiri KW Provenzano PP. Aligned collagen is a prognostic signature for survival in human breast carcinoma. Am J Pathol. (2011) 178:1221–32. 10.1016/j.ajpath.2010.11.076 21356373 PMC3070581

[19] Boyd NF Lockwood GA Byng JW Tritchler DL Yaffe MJ. Mammographic densities and breast cancer risk. Cancer Epidemiol Biomark Preven. (1998) 7:1133–44. 10.3233/BD-1998-103-412 9865433

[20] Boyd NF Guo H Martin LJ Sun L Stone J Fishell E. Mammographic density and the risk and detection of breast cancer. N Engl J Med. (2007) 356:227–36. 10.1056/NEJMoa062790 17229950

[21] Alowami S Troup S Al-Haddad S Kirkpatrick I Watson PH. Mammographic density is related to stroma and stromal proteoglycan expression. Br Cancer Res. (2003) 5:129–35. 10.1186/bcr622 12927043 PMC314426

[22] Mcconnell JC O'Connell OV Brennan K Weiping L Howe M Joseph L. Increased peri-ductal collagen micro- organization may contribute to raised mammographic density. Br Cancer Res. (2016) 18:5. 10.1186/s13058-015-0664-2 26747277 PMC4706673

[23] Acerbi I Cassereau L Dean I Shi Q Au A Park C. Human breast cancer invasion and aggression correlates with ECM stiffening and immune cell infiltration. Integr Biol. (2015) 7:1120–34. 10.1039/c5ib00040h 25959051 PMC4593730

[24] Huo CW Hill P Chew G Neeson PJ Halse H Williams ED. High Mammographic Density in Women Is Associated with Protumor Inflammation. Br Cancer Res. (2018) 20:1–16. 10.1186/s13058-018-1010-2 30092832 PMC6085707

[25] Provenzano PP Eliceiri KW Campbell JM Inman DR White JG Keely PJ. Collagen reorganization at the tumor-stromal interface facilitates local invasion. BMC Med. (2006) 4:38. 10.1186/1741-7015-4-38 17190588 PMC1781458

[26] Esbona K Yi Y Saha S Yu M Van Doorn RR Conklin MW. The presence of cyclooxygenase 2, tumor-associated macrophages, and collagen alignment as prognostic markers for invasive breast carcinoma patients. Am J Pathol. (2018) 188:616. 10.1016/j.ajpath.2017.10.025 29429545 PMC5963475

[27] Kessenbrock K Plaks V Werb Z. Matrix metalloproteinases: regulators of the tumor microenvironment. Cell. (2010) 141:52–67. 10.1016/j.cell.2010.03.015 20371345 PMC2862057

[28] Afik R Zigmond E Vugman M Klepfish M Shimshoni E Pasmanik-Chor M. Tumor macrophages are pivotal constructors of tumor collagenous matrix. J Exp Med. (2016) 213:2315–31. 10.1084/jem.20151193 27697834 PMC5068227

[29] Varol C. Tumorigenic interplay between macrophages and collagenous matrix in the tumor microenvironment. Methods Mol Biol. (2019) 1944:203–20. 10.1007/978-1-4939-9095-5_15 30840245

[30] Liu YL Lu Q Liang JW Xia Y Zhang W Hu BQ. High plasma fibrinogen is correlated with poor response to trastuzumab treatment in HER2 positive breast cancer. Medicine. (2015) 94:e481. 10.1097/MD.0000000000000481 25654390 PMC4602707

[31] Bae YK Kim A Kim MK Choi JE Kang SH Lee SJ. Fibronectin expression in carcinoma cells correlates with tumor aggressiveness and poor clinical outcome in patients with invasive breast cancer. Human Pathol. (2013) 44:2028–37. 10.1016/j.humpath.2013.03.006 23684510

[32] Hsieh JY Smith TD Meli VS Tran TN Botvinick EL Liu WF. Differential regulation of macrophage inflammatory activation by fibrin and fibrinogen. Acta Biomater. (2017) 47:14–24. 10.1016/j.actbio.2016.09.024 27662809 PMC5426227

[33] Sudhakaran PR Radhika A Jacob SS. Monocyte macrophage differentiation *in vitro*: fibronectin-dependent upregulation of certain macrophage-specific activities. Glycoconjugate J. (2007) 24:49–55. 10.1007/s10719-006-9011-2 17115276

[34] Mosesson MW. The role of fibronectin in monocyte/macrophage function. Progr Clin Biol Res. (1984) 154:155–75. 6089231

[35] Givant-Horwitz V Davidson B Reich R. Laminin-Induced Signaling in Tumor Cells. Cancer Lett. (2005) 223:1–10. 10.1016/j.canlet.2004.08.030 15890231

[36] Rick JW Chandra A Ore CD Nguyen AT Yagnik G Aghi MK. Fibronectin in malignancy: cancer-specific alterations, protumoral effects, and therapeutic implications. Semin Oncol. (2019) 46:284–90. 10.1053/j.seminoncol.2019.08.002 31488338 PMC6801036

[37] Provenzano PP Inman DR Eliceiri KW Knittel JG Yan L Rueden CT. Collagen density promotes mammary tumor initiation and progression. BMC Med. (2008) 6:11. 10.1186/1741-7015-6-11 18442412 PMC2386807

[38] Esbona K Inman D Saha S Jeffery J Schedin P Wilke L. COX-2 modulates mammary tumor progression in response to collagen density. Br Cancer Res. (2016) 18:35. 10.1186/s13058-016-0695-3 27000374 PMC4802888

[39] Linde N Casanova-Acebes M Sosa MS Mortha A Rahman A Farias E. Macrophages orchestrate breast cancer early dissemination and metastasis. Nat Commun. (2018) 9:21. 10.1038/s41467-017-02481-5 29295986 PMC5750231

[40] Leung E Xue A Wang Y Rougerie P Sharma VP Eddy R. Blood vessel endothelium-directed tumor cell streaming in breast tumors requires the HGF/C-met signaling pathway. Oncogene. (2017) 36:2680–92. 10.1038/onc.2016.421 27893712 PMC5426963

[41] Arwert EN Harney AS Entenberg D Wang Y Sahai E Pollard JW A unidirectional transition from migratory to perivascular macrophage is required for tumor cell intravasation. Cell Rep. (2018) 17:2445–59. 10.1016/j.celrep.2018.04.007 PMC5946803 29719241

[42] Hotchkiss KM Reddy GB Hyzy SL Schwartz Z Boyan BD Olivares-Navarrete R. Titanium surface characteristics, including topography and wettability, alter macrophage activation. Acta Biomater. (2016) 31:425–34. 10.1016/j.actbio.2015.12.003 26675126 PMC4728000

[43] Refai AK Textor M Brunette DM Waterfield JD. Effect of titanium surface topography on macrophage activation and secretion of proinflammatory cytokines and chemokines. J Biomed Mater Res Part A. (2004) 70:194–205. 10.1002/jbm.a.30075 15227664

[44] Tan KS Qian L Rosado R Flood PM Cooper LF. The role of titanium surface topography on J774A.1 macrophage inflammatory cytokines and nitric oxide production. Biomaterials. (2006) 27:5170–7. 10.1016/j.biomaterials.2006.05.002 16808973

[45] Hsieh JY Keating MT Smith TD Meli VS Botvinick EL Wendy Liu WF. Matrix crosslinking enhances macrophage adhesion, migration, and inflammatory activation. APL Bioengineering. (2019) 3:016103. 10.1063/1.5067301 31069336 PMC6481736

[46] Previtera ML Sengupta A. Substrate stiffness regulates proinflammatory mediator production through TLR4 activity in macrophages. PLoS ONE. (2015) 10:145813. 10.1371/journal.pone.0145813 26710072 PMC4692401

[47] Cao S Zhang X Edwards JP Mosser DM. NF-KappaB1. (P50) homodimers differentially regulate pro- and anti-inflammatory cytokines in macrophages. J Biol Chem. (2006) 281:26041–50. 10.1074/jbc.M602222200 16835236 PMC2642587

[48] McWhorter FY Wang T Nguyen P Chung T Liu WF. Modulation of macrophage phenotype by cell shape. Proc Natl Acad Sci USA. (2013) 110:17253–8. 10.1073/pnas.1308887110 24101477 PMC3808615

[49] Jain N Vogel V. Spatial confinement downsizes the inflammatory response of macrophages. Nat Mater. (2018) 17:1134–44. 10.1038/s41563-018-0190-6 30349032 PMC6615903

[50] Luu TU Gott SC Woo BWK Rao MP Liu WF. Micro- and nanopatterned topographical cues for regulating macrophage cell shape and phenotype. ACS Appl Mater Interf. (2015) 7:28665–72. 10.1021/acsami.5b10589 26605491 PMC4797644

[51] Wójciak-Stothard B Curtis A Monaghan W Macdonald K Wilkinson C. Guidance and activation of murine macrophages by nanometric scale topography. Exp Cell Res. (1996) 223:426–35. 10.1006/excr.1996.0098 8601420

[52] Giancotti FG Ruoslahti E. Integrin signaling. Science. (1999) 285:1028–33. 10.1126/science.285.5430.1028 10446041

[53] Ostermann G Weber KSC Zernecke A Schröder A Weber C JAM-I is a ligand of the B2 integrin LFA-I involved in transendothelial migration of leukocytes. Nat Immunol. (2002) 3:151–8. 10.1038/ni755 11812992

[54] Tian L Kilgannon P Yoshihara Y Mori K Gallatin WM Carpén O. Binding of T lymphocytes to hippocampal neurons through ICAM-5. (Telencephalin) and characterization of its interaction with the leukocyte integrin CD11a/CD18. Eur J Immunol. (2000) 30:810–18. 10.1002/1521-4141(200003)30:3<810::AID-IMMU810>3.0.CO;2-X 10741396

[55] Campanero MR Del Pozo MA Arroyo AG Sánchez-Mateos P Hernández-Caselles T Craig A. ICAM-3 interacts with LFA-1 and regulates the LFA-1/ICAM-1 cell adhesion pathway. J Cell Biol. (1993) 123:1007–16. 10.1083/jcb.123.4.1007 7901223 PMC2200154

[56] Wojcikiewicz EP Abdulreda MH Zhang X Moy VT. Force spectroscopy of LFA-1 and its ligands, ICAM-1 and ICAM-2. Biomacromolecules. (2006) 7:3188–95. 10.1021/bm060559c 17096550 PMC2570329

[57] Yakubenko VP Lishko VK Lam SCT Ugarova TP A molecular basis for integrin α M β 2 ligand binding promiscuity. J Biol Chem. (2002) 277:48635–42. 10.1074/jbc.M208877200 12377763

[58] Berton G Lowell CA. Integrin signalling in neutrophils and macrophages. Cell Signal. (1999) 11:621–35. 10.1016/S0898-6568(99)00003-0 10530871

[59] Altieri DC Agbanyo FR Plescia J Ginsberg MH Edgington TS Plow EF. A unique recognition site mediates the interaction of fibrinogen with the leukocyte integrin Mac-1. (CD11b/CD18). J Biol Chem. (1990) 265:12119–22. 1973686

[60] Lecoanet-Henchoz S Gauchat JF Aubry JP Graber P Life P Paul-Eugene N. CD23 regulates monocyte activation through a novel interaction with the adhesion molecules CD11b-CD18 and CD11c-CD18. Immunity. (1995) 3:119–25. 10.1016/1074-7613(95)90164-7 7621072

[61] Bilsland CA Diamond MS Springer TA. The leukocyte integrin p150,95. (CD11c/CD18) as a receptor for IC3b. Activation by a heterologous beta subunit and localization of a ligand recognition site to the I domain. J Immunol. (1994) 152:4582–89. 7512600

[62] Ihanus E Uotila LM Toivanen A Varis M Gahmberg CG. Red-Cell ICAM-4 is a ligand for the monocyte/macrophage integrin CD11c/CD18: characterization of the binding sites on ICAM-4. Blood. (2007) 109:802–10. 10.1182/blood-2006-04-014878 16985175

[63] Frick C Odermatt A Zen K Mandell KJ Edens H Portmann R Interaction of ICAM-1 with B2-Integrin CD11c/CD18: characterization of a peptide ligand that mimics a putative binding site on domain D4 of ICAM-1. Eur J Immunol. (2005) 35:3610–21. 10.1002/eji.200425914 16252253

[64] Sándor N Lukácsi S Ungai-Salánki R Orgován N Szabó B Horváth R. CD11c/CD18 dominates adhesion of human monocytes, macrophages and dendritic cells over CD11b/CD18. PLoS ONE. (2016) 11:e0163120. 10.1371/journal.pone.0163120 27658051 PMC5033469

[65] Ingalls RR Golenbock DT. CD11c/CD18, a transmembrane signaling receptor for lipopolysaccharide. J Exp Med. (1995) 181:1473–9. 10.1084/jem.181.4.1473 7535339 PMC2191975

[66] Choi J Leyton L Nham S. Characterization of AlphaX I-domain binding to thy-1. Biochem Biophys Res Commun. (2005) 331:557–61. 10.1016/j.bbrc.2005.04.006 15850796

[67] Gang J Choi J Lee JH Nham SU. Identification of critical residues for plasminogen binding by the AlphaX I-domain of the Beta2 integrin, AlphaXbeta2. Mol Cells. (2007) 24:240–6. 17978577

[68] Yakubenko VP Belevych N Mishchuk D Schurin A Lam SCT Ugarova TP. The role of integrin alpha D Beta2. (CD11d/CD18) in monocyte/macrophage migration. Exp Cell Res. (2008) 314:2569–78. 10.1016/j.yexcr.2008.05.016 18621369 PMC2621015

[69] Vieren MV Trong HL Wood CL Moore PF John T St Staunton DE. A novel leukointegrin, Adβ2, binds preferentially to ICAM-3. Immunity. (1995) 3:683–90. 10.1016/1074-7613(95)90058-6 8777714

[70] Yakubenko VP Yadav SP Ugarova TP. Integrin ADβ2, an adhesion receptor up-regulated on macrophage foam cells, exhibits multiligand-binding properties. Blood. (2006) 107:1643–50. 10.1182/blood-2005-06-2509 16239428 PMC1367263

[71] Hemler ME. VLA proteins in the integrin family: structures, functions, and their role on leukocytes. Annual Rev Immunol. (1990) 8:365–400. 10.1146/annurev.iy.08.040190.002053 2188667

[72] Bahou WF Potter CL Mirza M The VLA-2. (?2β1) I domain functions as a ligand-specific recognition sequence for endothelial cell attachment and spreading: molecular and functional characterization. Blood. (1994) 84:3734–41. 10.1182/blood.V84.11.3734.bloodjournal84113734 7949129

[73] Kamata T Puzon W Takada Y. Identification of putative ligand binding sites within I domain of integrin alpha 2 Beta 1. (VLA-2, CD49b/CD29). J Biol Chem. (1994) 269:9659–63. 7511592

[74] Chakraborty S Hu SY Wu SH Karmenyan A Chiou A. The interaction affinity between vascular cell adhesion molecule-1. (VCAM-1) and very late antigen-4. (VLA-4) analyzed by quantitative FRET. PLoS ONE. (2015) 10:e0121399. 10.1371/journal.pone.0121399 25793408 PMC4368157

[75] Hart R Greaves DR. Chemerin contributes to inflammation by promoting macrophage adhesion to VCAM-1 and fibronectin through clustering of VLA-4 and VLA-5. J Immunol. (2010) 185:3728–39. 10.4049/jimmunol.0902154 20720202

[76] Pytela R Pierschbacher MD Ruoslahti E. Identification and isolation of a 140 Kd cell surface glycoprotein with properties expected of a fibronectin receptor. Cell. (1985) 40:191–8. 10.1016/0092-8674(85)90322-8 3155652

[77] Schottelius M Laufer B Kessler H Wester HJ Ligands for mapping Avβ3-integrin expression *in vivo*. Accounts Chem Res. (2009) 42:969–80. 10.1021/ar800243b 19489579

[78] Shaw LM Lotz MM Mercurio AM. Inside-out integrin signaling in macrophages. Analysis of the role of the alpha 6A Beta 1 and Alpha 6B Beta 1 integrin variants in laminin adhesion by CDNA expression in an alpha 6 integrin-deficient macrophage cell line. J Biol Chem. (1993) 268:11401–8. 8496190

[79] Smith JW Cheresh DA Integrin. (?vβ3)-ligand interaction: identification of a heterodimeric RGD binding site on the vitronectin receptor. J Biol Chem. (1990) 265:2168–72. 1688848

[80] Kumawat AK Yu C Mann EA Schridde A Finnemann SC Mowat AM Expression and characterization of Avβ5 integrin on intestinal macrophages. Eur J Immunol. (2018) 48:1181–7. 10.1002/eji.201747318 29676784 PMC6207937

[81] Nandrot EF Anand M Almeida D Atabai K Sheppard D Finnemann SC Essential role for MFG-E8 as ligand for Avβ5 integrin in diurnal retinal phagocytosis. Proc Natl Acad Sci USA. (2007) 104:12005–10. 10.1073/pnas.0704756104 17620600 PMC1924559

[82] Smith JW Vestal DJ Irwin SV Burke TA Cheresh DA Purification and functional characterization of integrin αVβ5: an adhesion receptor for vitronectin. J Biol Chem. (1990) 265:11008–13. 1694173

[83] Schmid MC Khan SQ Kaneda MM Pathria P Shepard R Louis TL. Integrin CD11b activation drives anti-tumor innate immunity. Nat Commun. (2018) 9:5379. 10.1038/s41467-018-07387-4 30568188 PMC6300665

[84] Han C Jin J Xu S Liu H Li N Cao X. Integrin CD11b negatively regulates TLR-triggered inflammatory responses by activating syk and promoting degradation of MyD88 and TRIF via Cbl-B. Nat Immunol. (2010) 11:734–42. 10.1038/ni.1908 20639876

[85] Hu X Wang H Han C Cao X. Src promotes anti-inflammatory. (M2) macrophage generation via the IL-4/STAT6 pathway. Cytokine. (2018) 111:209–15. 10.1016/j.cyto.2018.08.030 30176559

[86] Erler JT Bennewith KL Cox TR Lang G Bird D Koong A. Hypoxia-induced lysyl oxidase is a critical mediator of bone marrow cell recruitment to form the premetastatic Niche. Cancer Cell. (2009) 15:35–44. 10.1016/j.ccr.2008.11.012 19111879 PMC3050620

[87] Wang Z Xiong S Mao Y Chen M Ma X Zhou X. Periostin promotes immunosuppressive premetastatic niche formation to facilitate breast tumour metastasis. J Patho. (2016) 239:484–95. 10.1002/path.4747 27193093

[88] Guo YP Martin LJ Hanna W Banerjee D Miller N Fishell E. Growth factors and stromal matrix proteins associated with mammographic densities. Cancer Epidemiol Biomark Prevent. (2001) 10:243–8. 11303594

[89] Curran CS Keely PJ. Breast tumor and stromal cell responses to TGF-β and hypoxia in matrix deposition. Matrix Biol. (2013) 32:95–105. 10.1016/j.matbio.2012.11.016 23262216 PMC3615133

[90] Cha BH Shin SR Leijten J Li YC Singh S Liu JC. Integrin-mediated interactions control macrophage polarization in 3D hydrogels. Adv Healthcare Mater. (2017) 6:21. 10.1002/adhm.201700289 28782184 PMC5677560

[91] Pakshir P Alizadehgiashi M Wong B Coelho NM Chen X Gong Z Dynamic fibroblast contractions attract remote macrophages in fibrillar collagen matrix. Nat Commun. (2019) 10:1850 10.1038/s41467-019-09709-6 31015429 PMC6478854

[92] Gowen BB Borg TK Ghaffar A Mayer EP. Selective adhesion of macrophages to denatured forms of type I collagen is mediated by scavenger receptors. Matrix Biol. (2000) 19:61–71. 10.1016/S0945-053X(99)00052-9 10686426

[93] Janabi M Yamashita S Hirano K Matsumoto K Sakai N Hiraoka H. Reduced adhesion of monocyte-derived macrophages from CD36-deficient patients to type I collagen. Biochem Biophys Res Commun. (2001) 283:26–30. 10.1006/bbrc.2001.4718 11322762

[94] Mazur A Holthoff E Vadali S Kelly T Post SR. Cleavage of type I collagen by fibroblast activation protein-α enhances class a scavenger receptor mediated macrophage adhesion. PLoS ONE. (2016) 11:e0150287. 10.1371/journal.pone.0150287 26934296 PMC4774960

[95] Yakubenko VP Bhattacharjee A Pluskota E Cathcart MK. Amβ2 integrin activation prevents alternative activation of human and murine macrophages and impedes foam cell formation. Circulation Res. (2011) 108:544–54. 10.1161/CIRCRESAHA.110.231803 21252155 PMC3080038

[96] Hagemann T Wilson J Burke F Kulbe H Li NF Plüddemann A. Ovarian cancer cells polarize macrophages toward a tumor-associated phenotype. J Immunol. (2006) 176:5023–32. 10.4049/jimmunol.176.8.5023 16585599

[97] Neyen C Plüddemann A Mukhopadhyay S Maniati E Bossard M Gordon S. Macrophage scavenger receptor A promotes tumor progression in murine models of ovarian and pancreatic cancer. J Immunol. (2013) 190:3798–805. 10.4049/jimmunol.1203194 23447685 PMC3608578

[98] Antonov AS Antonova GN Munn DH Mivechi N Lucas R Catravas JD AVβ3 integrin regulates macrophage inflammatory responses via PI3 kinase/Akt-dependent NF-?B activation. J Cell Physiol. (2011) 226:469–76. 10.1002/jcp.22356 20672329 PMC3235728

[99] Evans R Flores-Borja F Nassiri S Miranda E Lawler K Grigoriadis A. Integrin-mediated macrophage adhesion promotes lymphovascular dissemination in breast cancer. Cell Rep. (2019) 27:1967–78.e4. 10.1016/j.celrep.2019.04.076 31091437 PMC6527923

[100] Digiacomo G Tusa I Bacci M Cipolleschi MG Sbarba PD Rovida E. Fibronectin induces macrophage migration through a SFK-FAK/CSF-1R pathway. Cell Adhesion Migrat. (2017) 11:327–37. 10.1080/19336918.2016.1221566 27588738 PMC5569968

[101] Pollard JW. Macrophages define the invasive microenvironment in breast cancer. J Leukocyte Biol. (2008) 84:623–30. 10.1189/jlb.1107762 18467655 PMC2516896

[102] Zajd CM Ziemba AM Miralles GM Nguyen T Feustel PJ Dunn SM Bone marrow-derived and elicited peritoneal macrophages are not created equal: the questions asked dictate the cell type used. Front Immunol. (2020) 11:269 10.3389/fimmu.2020.00269 32153579 PMC7047825

[103] Ng AYH Tu C Shen S Xu D Oursler MJ Qu J. Comparative characterization of osteoclasts derived from murine bone marrow macrophages and RAW 264.7 cells using quantitative proteomics. JBMR Plus. (2018) 2:328–40. 10.1002/jbm4.10058 30460336 PMC6237207

[104] Schildberger A Eva R Tanja E Katharina S Viktoria W. Monocytes, peripheral blood mononuclear cells, and THP-1 cells exhibit different cytokine expression patterns following stimulation with lipopolysaccharide. Mediat Inflamm. (2013). 2013:697972. 10.1155/2013/697972 23818743 PMC3681313

[105] Shiratori H Feinweber C Luckhardt S Linke B Resch E Geisslinger G. THP-1 and human peripheral blood mononuclear cell-derived macrophages differ in their capacity to polarize *in vitro*. Mol Immunol. (2017) 88:58–68. 10.1016/j.molimm.2017.05.027 28600970

[106] Ingersoll MA Spanbroek R Lottaz C Gautier EL Frankenberger M Hoffmann R. Comparison of gene expression profiles between human and mouse monocyte subsets. Blood. (2010) 115:e10–9. 10.1182/blood-2009-07-235028 19965649 PMC2810986

[107] Martinez FO Helming L Milde R Varin A Melgert BN Draijer C. Genetic programs expressed in resting and IL-4 alternatively activated mouse and human macrophages: similarities and differences. Blood. (2013) 121:e57–69. 10.1182/blood-2012-06-436212 23293084

[108] Spiller KL Wrona EA Romero-Torres S Pallotta I Graney PL Witherel CE. Differential gene expression in human, murine, and cell line-derived macrophages upon polarization. Exp Cell Res. (2016) 347:1–13. 10.1016/j.yexcr.2015.10.017 26500109

[109] Li Y Liu TM. Discovering macrophage functions using *in vivo* optical imaging techniques. Front Immunol. (2018) 9:502. 10.3389/fimmu.2018.00502 29599778 PMC5863475

[110] Rostam HM Paul MR Morgan R Alexander N Amir M. Image based machine learning for identification of macrophage subsets. Sci Rep. (2017) 7:3521. 10.1038/s41598-017-03780-z 28615717 PMC5471192

[111] Rebelo SP Pinto C Martins TR Harrer N Estrada MF Loza-Alvarez P. 3D-3-culture: a tool to unveil macrophage plasticity in the tumour microenvironment. Biomaterials. (2018) 163:185–97. 10.1016/j.biomaterials.2018.02.030 29477032

[112] Irimia D Wang X. Inflammation-on-a-chip: probing the immune system *ex vivo*. Trends Biotechnol. (2018) 36:923–7. 10.1016/j.tibtech.2018.03.011 29728272 PMC6098972

[113] Boussommier-Calleja A Li R Chen MB Wong SC Kamm RD. Microfluidics: a new tool for modeling cancer–immune interactions. Trends Cancer. (2016) 2:6–19. 10.1016/j.trecan.2015.12.003 26858990 PMC4743529

[114] Meshik X O'Neill PR Gautam N. Physical plasma membrane perturbation using subcellular optogenetics drives integrin-activated cell migration. ACS Synthetic Biol. (2019) 8:498–510. 10.1021/acssynbio.8b00356 30764607 PMC7081452

[115] Pertz O Hodgson L Klemke RL Hahn KM. Spatiotemporal dynamics of RhoA activity in migrating cells. Nature. (2006) 440:1069–72. 10.1038/nature04665 16547516

[116] Dagliyan O Tarnawski M Chu PH Shirvanyants D Schlichting I Dokholyan NV. Engineering extrinsic disorder to control protein activity in living cells. Science. (2016) 354:1441–4. 10.1126/science.aah3404 27980211 PMC5362825

[117] Coste A Oktay MH Condeelis JS Entenberg D. Intravital imaging techniques for biomedical and clinical research. Cytometry. (2019) 97:448–57. 10.1002/cyto.a.23963 31889408 PMC7210060

[118] Kedrin D Gligorijevic B Wyckoff J Verkhusha VV Condeelis J Segall JE. Intravital imaging of metastatic behavior through a mammary imaging window. Nat Methods. (2008) 5:1019–21. 10.1038/nmeth.1269 18997781 PMC2820719

[119] Szulczewski JM Inman DR Entenberg D Ponik SM Aguirre-Ghiso J Castracane J. *In vivo* visualization of stromal macrophages via label-free FLIM-based metabolite imaging. Sci Rep. (2016) 6:25086. 10.1038/srep25086 27220760 PMC4879594

[120] Schreiber HA Loschko J Karssemeijer RA Escolano A Meredith MM Mucida D. Intestinal monocytes and macrophages are required for T cell polarization in response to citrobacter rodentium. J Exp Med. (2013) 210:2025–39. 10.1084/jem.20130903 24043764 PMC3782042

[121] Harney AS Arwert EN Entenberg D Wang Y Guo P Qian BZ. Real-time imaging reveals local, transient vascular permeability, and tumor cell intravasation stimulated by TIE2hi macrophage–derived VEGFA. Cancer Discov. (2015) 5:932–43. 10.1158/2159-8290.CD-15-0012 26269515 PMC4560669

[122] Entenberg D Voiculescu S Guo P Borriello L Wang Y Karagiannis GS. A permanent window for the murine lung enables high-resolution imaging of cancer metastasis. Nat Methods. (2018) 15:73–80. 10.1038/nmeth.4511 29176592 PMC5755704

[123] Berezin MY Achilefu S. Fluorescence lifetime measurements and biological imaging. Chem Rev. (2010) 110:2641–84. 10.1021/cr900343z 20356094 PMC2924670

[124] Lakowicz JR Szmacinski H Nowaczyk K Johnson ML. Fluorescence lifetime imaging of free and protein-bound NADH. Biochemistry. (1992) 89:1271–5. 10.1073/pnas.89.4.1271 1741380 PMC48431

[125] Alfonso-García A Smith TD Datta R Luu TU Gratton E Potma EO. Label-free identification of macrophage phenotype by fluorescence lifetime imaging microscopy. J Biomed Optics. (2016) 21:046005. 10.1117/1.JBO.21.4.046005 27086689 PMC4833856

[126] Qiu S Deng L Liao X Nie L Qi F Jin K. Tumor-associated macrophages promote bladder tumor growth through PI3K/AKT signal induced by collagen. Cancer Sci. (2019) 110:2110–8. 10.1111/cas.14078 31120174 PMC6609800

[127] Covarrubias AJ Aksoylar HI Horng T. Control of macrophage metabolism and activation by MTOR and Akt Signaling. Semin Immunol. (2015). 27:286–96. 10.1016/j.smim.2015.08.001 26360589 PMC4682888

